# Clinical Outcomes of Endovascular Treatment for Carotid Artery Dissection Without Intracranial Large Vessel Occlusion in Patients With Cerebral Ischemia Presentation

**DOI:** 10.3389/fneur.2021.713190

**Published:** 2022-02-02

**Authors:** Joong-Goo Kim, Chul-Hoo Kang, Jay Chol Choi, Yunsun Song, Dae Chul Suh, Deok Hee Lee

**Affiliations:** ^1^Department of Neurology, Jeju National University Hospital, School of Medicine, Jeju National University, Jeju, South Korea; ^2^Department of Radiology, Asan Medical Center, University of Ulsan College of Medicine, Seoul, South Korea

**Keywords:** carotid artery, internal, dissection, ischemic stroke, stents, angioplasty, balloon

## Abstract

**Background and Purpose:**

We describe the clinical characteristics and outcomes (including the long-term patency of endovascular treatment [EVT]) of patients with acute ischemic strokes (AISs) featuring carotid artery dissection (CAD) but not intracranial large vessel occlusion.

**Methods:**

We retrospectively reviewed patients who underwent EVT for spontaneous or post-traumatic AISs with CAD over a 13 year period from September 2005 to November 2018. The indications for EVT in patients with AIS-related CAD were a pretreatment diffusion-weighted imaging-Alberta Stroke Program early computed tomography (ASPECT) score > 6 and, clinical-diffusion mismatch. But, if the patients showed fluctuated ischemic symptoms, the joint decision by a stroke neurologist and neurointerventionist was done according to the onset-to-door time, symptoms, patient data, and the initial neuroimaging findings whether indicated that EVT was appropriate.

**Results:**

Twenty-two dissected carotid arteries underwent balloon angioplasty and/or stent placement. The patients were 6 women and 16 men of median age 46 years. Twelve lacked any trauma history. Recombinant tissue plasminogen activator was prescribed for two (9.1%) patients. Four developed symptomatic intracranial hemorrhages (18.2%) but 86.4% exhibited modified Rankin scores ≤ 2.

**Conclusions:**

Although attention to the hemorrhagic complication is required, EVT for selective patients with cerebral ischemia associated with CAD may be safe and acceptable treatment strategy for reconstruction of luminal patency, with good clinical outcomes. Prospective large-scale randomized studies are required to optimize EVT for CAD patients.

## Introduction

Recent trials of endovascular treatment (EVT) have proven its effectiveness in patients with acute ischemic stroke (AIS) and intracranial large-vessel occlusion (ILVO) compromising anterior cerebral circulation (1). Carotid artery dissection is a rare cause of ischemic stroke, but is responsible for 20–25% of strokes in young patients (2). The cerebral ischemia associated with CAD reflects embolisms of dissected vessels, and triggers hemodynamic insufficiency (3). CAD with concomitant ILVO is usually associated with very poor clinical outcomes; emergency EVT is required (4). However, conservative treatment is an option if the ischemic symptoms associated with CAD (without ILVO) are mild and do not progress significantly (5). However, CAD patients without ILVO may develop hemodynamic insufficiency or a recurrent embolism raising a strong suspicion of ischemic symptom fluctuation (6). Although most patients with this AIS subtype undergo EVT, the clinical outcomes have been but rarely studied. As EVT timing depends on the symptoms, identification of the available time window for patients with CAD without ILVO remains challenging. Here, we share our clinical experience with, and the results of, management of AIS related to CAD, and explore EVT safety and efficacy in patients with CAD without ILVO.

## Methods

### Study Population

We reviewed the data on consecutive patients with CAD evaluated in our comprehensive stroke center between January 1 September 2005 and November 31, 2018. All were retrospectively selected from a prospective neurointerventional database and a stroke registry. Clinical and radiological data were reviewed. We collected information on patient demographics, vascular risk factors, imaging findings, time from symptom onset to the procedure, baseline National Institutes of Health Stroke Scale (NIHSS) scores, the modified Rankin Scale (mRS) scores at 3 months, and the length of hospital stay. Cerebral angiograms were reviewed in terms of the location of dissection and reperfusion status after EVT. The inclusion criteria of EVT were as follows: (1) Patients with AIS associated CAD which showed clinical-diffusion mismatch or symptom fluctuation [at least pretreatment Diffusion-Weighted Imaging–Alberta Stroke Program Early Computed Tomography Scores (DWI-ASPECT) > 6] and (2) if patients who showed fluctuated ischemic symptoms were judged to be beneficial to performing EVT for CAD by a discussion between neurologist and neurointerventionist about onset-to-door time, symptoms, patient information, and initial neuro-imaging findings. We excluded patients when (1) incidentally identified CAD (thus lacking clinical symptoms), (2) those with onset to puncture time >1 week, (3) DWI-ASPECT ≤ 6, (4) those who underwent mechanical thrombectomy to treat ILVO, and (5) those with an intracerebral hemorrhage evident in initial CT or magnetic resonance imaging (MRI). The histories of all ischemic events were recorded, and the physical and neurological statuses of all patients were evaluated by our stroke neurologists. In line with our acute stroke management protocol, all patients underwent MRI with magnetic resonance angiography (MRA) or CT angiography (CTA) of the circle of Willis and the carotid vessels prior to EVT. Detailed EVT data were retrieved from our electronic medical records and our picture archive and communication system (PACS). This study was approved by our institutional review board, and the need for written, informed patient consent was waived because of the retrospective nature of the study.

### Interventions

All procedures were performed via the percutaneous transfemoral route with patients under local anesthesia. All EVT procedures were performed by two of our neurointerventionalists who treat all patients with symptomatic CAD. After placement of a sheath introducer, unfractionated heparin was intravenously administered to maintain the activated clotting time at 2-fold the normal value. All patients were prescribed dual antiplatelet agents and a statin before the procedure (7). Typically, a long 6–8 Fr. sheath (Shuttle-SL; Cook Medical, Bloomington, IN, USA) was deployed. A microcatheter of internal diameter 0.021 or 0.027 inches was navigated distally to the point of dissection, over a 0.014-inch steerable microwire. When the microcatheter lay above the dissected carotid artery, angiography was performed to identify the arterial lumen. Then the microcatheter was replaced with a 300 cm microwire for delivery of a balloon or a stent catheter. Balloon angioplasty with stent placement allowed of reperfusion in selected patients. Balloon angioplasty was performed at the discretion of the neurointerventionist.

### Imaging

All patients underwent MRI with magnetic resonance angiography (MRA) or CT angiography (CTA) of the circle of Willis and the carotid vessels. A final diagnosis of CAD was based on prolonged conventional angiography performed using an adequate contrast level. Imaging follow-up performed within 72 h after EVT comprised three-dimensional time-of-flight MRI and CTA, including T2^*^imaging, fluid-attenuated inversion recovery (FLAIR), and diffusion imaging.

### Classification of CAD

**Figure 1:**
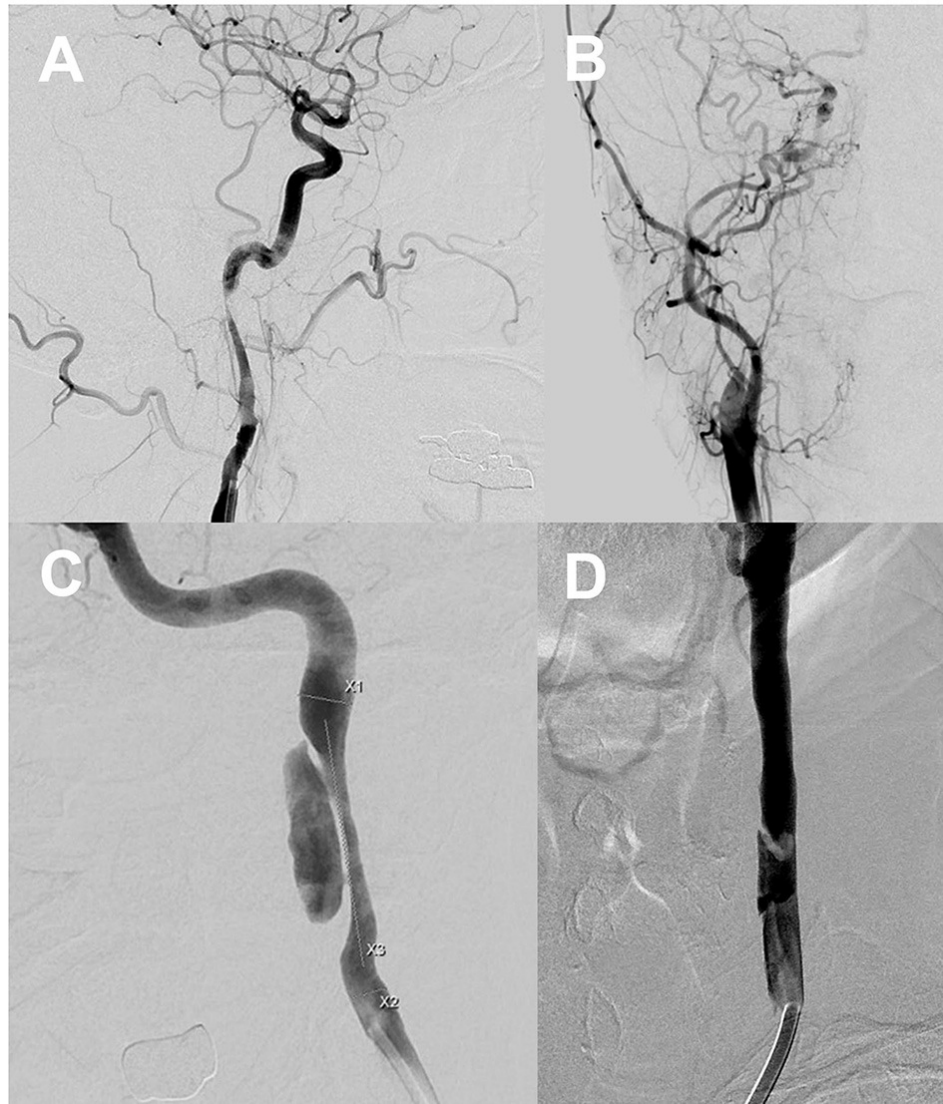
Type I dissections with intact intima **(A,B)**. The Type IA dissection (left) exhibits sustained antegrade flow without complete occlusion. The Type IB dissection (right) is completely occluded. Type II dissections with intimal disruptions **(C,D)**. The Type IIA dissection **(C)** exhibits a small intimal disruption with a side-wall aneurysm. The Type IIB dissection **(D)** exhibits a clear intimal flap and an aneurysmal dilation. X1: Distal carotid luminal diameter, X2: Proximal carotid luminal diameter, X3: Length of a dissected segment of the carotid artery.

We used the Borgess classification (8) of CAD that reflects the intimal tear status of the dissected vessel and its influence on blood flow assessed via digital subtraction imaging (Figure 1). Intimal injury was considered present if imaging revealed contrast filling outside the vascular lumen, a false lumen with an intimal flap, or fusiform vessel dilation. We explored whether blood attained the carotid artery beyond the dissecting segment. Type I dissections featured intact intimae, and type II dissections had injured intimae; both types were divided into two subtypes. Type IA dissections exhibited luminal stenosis caused by an intramural hematoma, but antegrade flow was preserved. Type IB dissections evidenced no antegrade flow. Type IIA dissections exhibited small, focal intimal tears; one intimal side became filled with contrast medium and stagnation was evident within the dissection. Type IIB dissections featured intimal flaps with false lumina distinct from the normal lumina, or aneurysmal dilation of the dissected vessels. The location of carotid dissection was recorded according to the initial DSA.

### Outcomes

Neurological status after EVT was evaluated by dedicated neurologists; all patients were transferred to the neurological intensive care unit after EVT. Clinical outcomes were assessed immediate improvement of NIHSS after interventional therapy, and via modified Rankin Scale (mRS) scoring at 90 days; scores ≤ 2 indicated functional independence and good clinical outcomes. Patients exhibiting increases >2 points on the NIHSS underwent CT or MRI, with the exception of those except for whom these procedures were contraindicated or whose cooperation was poor. We evaluated 3 month mortality, the length of hospital stay, cerebral hemorrhage status (any hemorrhagic transformation or subarachnoid hemorrhage evident on follow-up images), symptomatic intracranial hemorrhage status (any parenchymal hematoma, subarachnoid hemorrhage, or intraventricular hemorrhage associated with worsening of the NIHSS score by ≥4 points within 24 h of EVT) (9), postprocedural infarct extension, and any newly detected infarction. Stent patency was assessed via ultrasound, CTA, or MRA within the 3 days, at the discretion of the attending neurologist.

## Results

**Figure 2:**
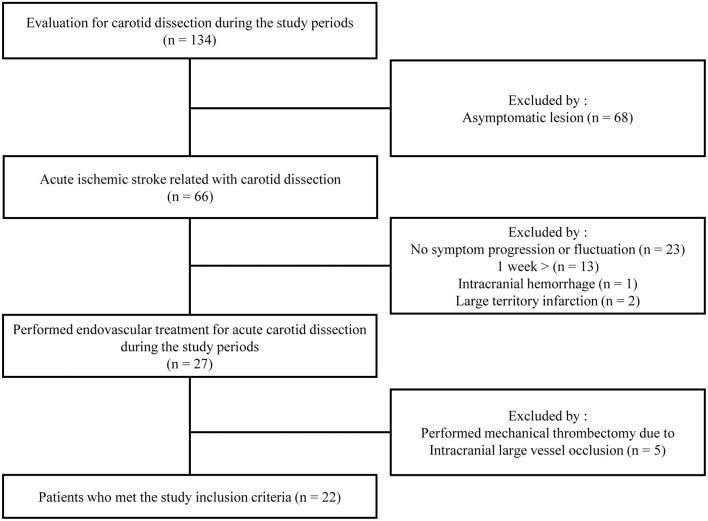
Flowchart of the patient selection process.

A patient flowchart is shown in Figure 2. Patient baseline characteristics are summarized in Tables 1, 2. All patients exhibited small ischemic cores as indicated by ASPECT scores > 6 in initial non-contrast CT or evidence of an overt diffusion/perfusion mismatch. Twenty two patients met the inclusion criteria (Figure 2). The median age was 46 years (interquartile range [IQR] 42.0–60.0 years) and 15 (57.7%) were men. The median interval from symptom onset to a procedure was 53.7 h (IQR 18.3–72.0 h) (Table 1). Ten (45.5%) trauma patients had high-energy non-penetrating injuries, and five (22.7%) headaches associated with the initial ischemic symptoms. Two (9.1%) patients had experienced previous strokes, but all patients had baseline mRS scores of 0. Tissue plasminogen activator was administered to two (9.1%) patients. All stents were proved the patency at follow-up examination within the 3 days after EVT.

**Table 1:** Baseline patient characteristics.

	**(*N* = 22)**
**Demographics**	
Age, years	46.0 (42.0–60.0)
Sex, male	15 (68.2%)
**Comorbidities and risk factors**
Hypertension	6 (27.3%)
Diabetes mellitus	1 (4.5%)
Hypercholesterolemia	3 (13.6%)
Atrial fibrillation	1 (4.5%)
Coronary artery disease	2 (9.1%)
Previous stroke	2 (9.1%)
Cancer	0 (0%)
Smoking	8 (36.4%)
Alcohol consumption	10 (45.5%)
Trauma	10 (45.5%)
Headache	5 (22.7%)
**Borgess classification**
IA	8 (36.4%)
IB	7 (31.8%)
IIA	5 (22.7%)
IIB	2 (9.1%)
Tissue plasminogen activator	2 (9.1%)
Initial NIHSS	1.0 (0.0–6.0)

*NIHSS, national institutes of health stroke scale*.

**Table 2:** Clinical and imaging outcomes.

	**(*N* = 22)**
Any hemorrhage evident on follow-up imaging	4 (18.2%)
HI-1	0 (0.0%)
HI-2	0 (0.0%)
PH-1	1 (4.5%)
PH-2	3 (13.6%)
Symptomatic hemorrhage	4 (18.2%)
NIHSS at discharge	1.0 (0.0–3.0)
mRS at 90 days	
0	10 (45.5%)
1	7 (31.8%)
2	2 (9.1%)
4	3 (13.6%)
Mortality at 90 days	0 (0.0%)
Infarct volume extension on follow-up imaging	6 (27.3%)
Clinical worsening within 24hr of the procedure	4 (18.2%)
Hospital stay, days	9.0 (8.0–11.0)
Craniectomy	2 (9.1%)
Periprocedural in-stent thrombosis	1 (4.5%)

*HI, hemorrhagic infarction; PH, parenchymal hematoma; NIHSS, national institutes of health stroke scale; mRS„ modified rankin scale*.

In terms of the Borgess classification, eight (36.4%) patients exhibited diffuse luminal narrowing without intimal disruption (Type IA), and seven (31.8%) had carotid occlusion without residual anterograde flow (Type IB). Five (22.7%) progressed to small intimal disruptions with side-wall aneurysms and two (9.1%) evidenced clear intimal flaps. The location of carotid dissection was described in Table 3. Multiple stents were placed (median of 1.2 per patient) in the distal intracranial lesions, including self-expanding carotid stents (15/22 patients), coronary balloon-expanding stents (3/22 patients), and self-expanding intracranial stents (5/15 patients). Long-segment, carotid stent reconstruction was technically successful in all patients with no significant (50%) residual stenosis/occlusion or flow limitation evident in post-procedural angiographic analyses.

**Table 3:** EVT for symptomatic patients with CAD.

**ID**	**Age**	**Type**	**Side**	**Symptom**	**Trauma**	**Location**	**NIHSS**	**IVtPA**	**LSCW to procedure time**	**Infarct pattern**	**Hemodynamic insufficiency**	**Embolism**	**Infarct volume extension on follow-up imaging**	**Clinical worsening within 24 h of the procedure**	**Hemorrhage**	**Stent**	**mRS at 90 days**
1	79	1	Left	Aphasia, Neck pain	No	C2-4	21	No	8 h	Cortical/border zone	Yes	Yes	Yes	No	No	PRECISE	4
2	48	2	Left	Dysarthria	No	Petrous-C2	1	No	72 h	Cortical/border zone	Yes	Yes	No	Yes	PH-2	PRECISE	1
3	42	2	Left	Aphasia	No	C3-C4	7	No	24 h	Cortical/border zone	Yes	Yes	Yes	Yes	No	Neuroform	4
4	47	1	Left	Right-side weakness	No	C3-C4	6	Yes	11 h	Cortical/border zone	Yes	Yes	No	No	No	PRECISE	1
5	45	2	Left	Aphasia, Right-side weakness	No	Petroug-C3	16	No	15 h	Deep/border zone	Yes	Yes	No	No	No	PRECISE	1
6	77	1	Left	Right-side weakness	Yes	C6-7	1	No	72 h	Cortical/border zone	Yes	No	No	No	No	PRECISE	0
7	40	2	Left	Visual disturbance	Yes	Cavernous-Petrous	4	No	72 h	Cortical/border zone	Yes	Yes	No	No	No	Driver	1
8	43	2	Left	Dysarthria	No	C2-C4	0	No	72 h	No lesion	Yes	Yes	No	No	No	Wallstent	0
9	70	2	Left	Aphasia	No	C2-C4	16	Yes	7 h	Cortical/border zone	Yes	Yes	Yes	No	No	Solitaire FR	1
10	41	2	Left	Right-side weakness	Yes	Petrous	0	No	1 week	Cortical/border zone	Yes	Yes	No	No	No	Neuroform	0
11	54	1	Left	Aphasia	Yes	Petrous-C1	4	No	53 h	Cortical/border zone	Yes	Yes	No	No	No	Neuroform	1
12	33	2	Left	Mono-ocular blindness	Yes	C2-C3	2	No	48 h	No lesion	Yes	No	No	No	No	Protégé	0
13	50	2	Left	Right-hand weakness	Yes	Petrous-C3	0	No	1 week	Cortical/border zone	Yes	Yes	No	No	No	Xpert	0
14	43	2	Right	Mono-ocular blindness, Right	Yes	C2-C4	0	No	1 week	No lesion	Yes	No	Yes	Yes	PH-2	PRECISE	2
15	45	1	Both	Visual disturbance, headache	Yes	C2-C3	0	No	1 week	No lesion	Yes	No	No	No	No	Acculink	0
16	85	2	Right	Confusion	Yes	C1-C2	1	No	19 h	Cortical/borderzone	Yes	Yes	No	No	No	Protégé	1
17	42	2	Right	Left-side weakness	No	C1-4	0	No	12 h	Cortical	Yes	Yes	No	No	No	Protégé	0
18	40	1	Right	Mono-ocular blindness	No	C2-C3	0	No	25 h	No lesion	Yes	No	No	No	No	Xpert	0
19	61	2	Left	Dysarthria	No	C1-C3	14	No	24 h	Deep/borderzone	Yes	Yes	No	Yes	PH-2	PRECISE	4
20	48	2	Left	Seizure	Yes	C1-3	0	No	24 h	No lesion	Yes	No	No	No	No	Protégé	0
21	45	2	Right	Visual disturbance	No	Petrous-C3	0	No	16 h	No lesion	Yes	No	Yes.	No	No	LVIS Blue	0
22	60	1	Left	Dysarthria, Visual disturbance	No	C2-C3	3	No	24h	Cortical/borderzone	Yes	Yes	Yes	No	PH-1	PRECISE	2

*NIHSS, national institutes of health stroke scale; LCSW, last significant clinical worsening; mRS, modified rankin scale; PH, parenchymal hematoma*.

### Clinical Outcomes

**Figure 3:**
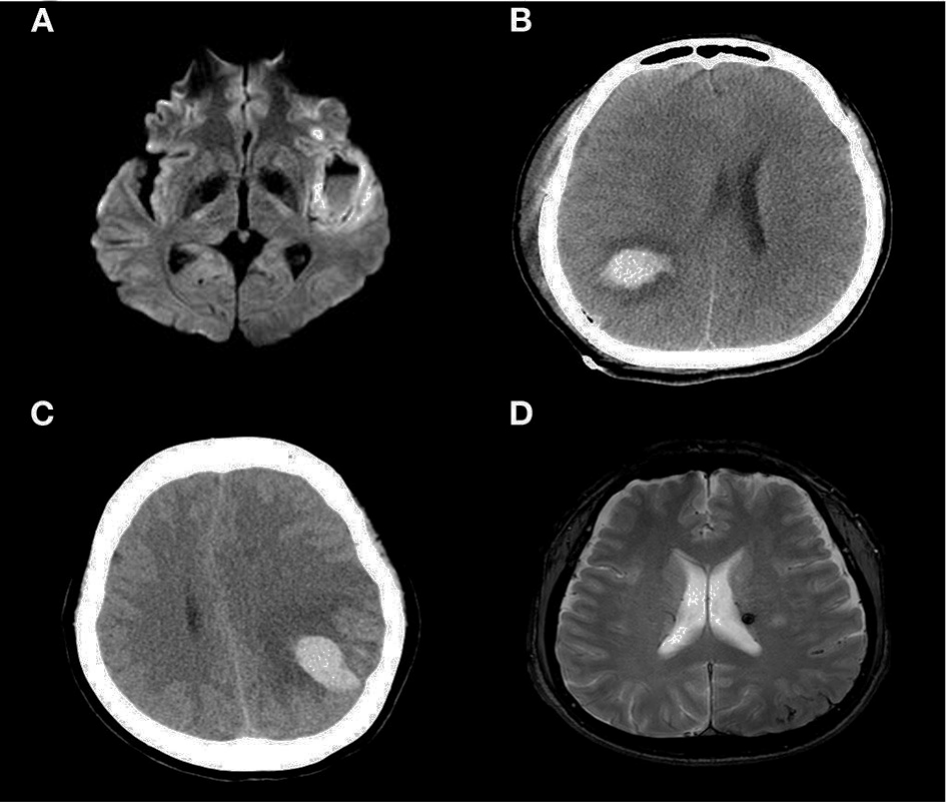
Diffusion-weighted image showing a large, left-side, insular cortical hemorrhage with a distinct fluid level **(A)**. A CT scan showing a parenchymal hemorrhage in the right temporal-parietal area **(B)** and left parietal cortex **(C)**. A newly developed (small) subarachnoid hemorrhage in the left parietal convexity and microbleeds in the thalamus **(D)**.

Procedural complications developed in four (18.2%) patients (1, 13, 16, and 17); all developed symptomatic hemorrhage (parenchymal hematoma grades 1 or 2 using the European Cooperative Acute Stroke Study criteria) in the ipsilateral hemisphere secondary to reperfusion/hyperperfusion injury. Although no procedure-related mortality was noted to discharge or the 90 day follow-up, subsequent clinical deterioration and a poor clinical outcome at 90 days yielded an overall procedural morbidity of three (13.6%). Rapid post-procedural clinical improvement was observed; the median NIHSS score of 1.0 (0.0–6.0) at admission became 1.0 (0.0–3.0) at discharge. At follow-up during admission, 6 (27.3%) patients demonstrated the infarct volume increased at follow-up imaging and all of those with symptomatic hemorrhage worsened within 24 h (Figure 3). Two (9.1%) patients underwent decompressive hemicraniectomy after their procedures and one (4.5%) developed an in-stent thrombosis (Table 2). There were no recurrent ischemic symptoms or strokes during clinical follow-up. Follow-up carotid Doppler ultrasound and CTA/digital subtraction angiography data collected 3–6 months later were available for 21/22 patients, of whom 21 evidenced stent patency, complete restoration of the carotid artery caliber, and no evidence of in-stent thrombosis or significant re-stenosis, suggestive of successful stent-associated flow diversion and intimal flap reconstruction (Table 3).

## Discussion

We report the case series of flow-limiting CADs without ILVO, presenting as AIS requiring EVT. Most CAD patients who underwent EVT in our report exhibited favorable clinical outcomes and successful revascularization. The median discharge NIHSS score was 1.0 (0.0–3.0) and the 90 day mRS scores were good for 86.4% of those who were so scored. We wished to evaluate the outcomes of EVT in patients with ischemic symptoms caused by the hemodynamic deficit associated with CAD alone. EVT is not always necessary, as noted in an earlier studies (10), EVT can serve as a rescue therapy for CAD patients who lack adequate cerebral perfusion (4, 6). Restoration of antegrade flow to the brain parenchyma is critical when treating hemodynamically unstable CAD; this prevents recurrent thromboembolism and re-occlusion after successful recanalization (11). In addition, EVT for CAD patients not only restores anterograde flow but also inhibit the formation of new thrombi under the torn vessel wall because the flying intima becomes attached to the sidewall (12).

We graded patients using the Borgess classification (8), which is based on the presence or absence of a ruptured tunica intima in the dissected vessel, and the hemodynamic effects on blood flow, as revealed by conventional angiography. However, our patient number was small, and differences in prognosis by the Borgess classification could not be determined.

Angioplasty and stenting seek to improve perfusion by closing a false lumen and restoring the patency of the injured vessel. However, two major risk factors are in play: iatrogenic CAD expansion and reperfusion injury. The device must pass through the dissected carotid artery, and it is always possible that the wire or catheter may enter the false lumen. This can be very dangerous if anterograde flow is lacking, as in Borgess Type IB patients. We encountered no iatrogenic injuries. Reperfusion injury is caused by abrupt restoration of cerebral blood flow following revascularization (13), and may trigger the loss of flow autoregulation followed by damage to the blood–brain barrier (14). Of all patients, 18.2% developed symptomatic hemorrhages (reperfusion injury). Craniectomy was performed in 50% of these cases. All patients with reperfusion injuries had undergone the procedure more than 24 h after symptom onset. Re-perfusion has been reported deep associated with the provocation of hemorrhagic transformation through infarcted lesions. Previous reports based on MRI stated that blood-brain barrier disruption was an independent predictor of hemorrhagic transformation and reperfusion at the ischemic core was the most significant independent predictor of early blood-brain barrier disruption. Also, such injury may be related to the use of dual antiplatelet agents to prevent in-stent thrombosis.

No well-designed clinical trial has explored the optimal peri-procedural management of patients with CAD. Although we excluded patients with ILVOs, symptomatic hemorrhagic transformation or parenchymal hematoma (known complications of reperfusion injury) may develop peri-procedurally even after extracranial EVT (15). Prevention of reperfusion injury is most important; physicians must be aware that CAD patients are at risk for such injury during the entire admission period (13). All patients who undergo EVT require intensive hemodynamic monitoring; reperfusion injury requires prompt diagnosis and management (16). In our view, the optimal therapeutic approach is vigilant monitoring of vital signs and control of the systolic blood pressure (17). Although we do not have enough clinical evidence supporting such an approach, we believe that it greatly reduces the risk of potentially devastating reperfusion injury. In our four patients with reperfusion injuries (intracranial hemorrhages), we controlled the arterial pressures within hours; the several days without antiplatelet agents did not precipitate in-stent thrombosis.

Our EVT procedure differed from that employed to treat atherosclerotic stenosis. In our patients, the lesions were considerably higher and/or extended, and the dissected segment requiring treatment was longer than atherosclerosis (18). However, in most patients, if the entry zone of the dissected intima is fully covered, a 40 mm stent is sufficient. Stents were initially placed after balloon angioplasty, but in later cases, angiography revealed luminal patency even after stenting alone. We did not employ an embolic protective device because of the risk that the dissection might expand; we encountered no distal embolization.

The limitations of this study include its retrospective cross-sectional nature. The number of patients was small and we lacked a control group. However, we provide preliminary data that may guide future prospective randomized studies seeking to confirm our results and to consolidate a therapeutic approach for the management of ischemic strokes related to CAD.

We found that EVT of ischemic strokes associated with CAD afforded an acceptable reperfusion rate and good outcomes. Further studies are necessary to validate our findings and to explore their clinical implications with regard to triggering additional and timely interventions in patients with CAD.

## Data Availability Statement

The original contributions presented in the study are included in the article/supplementary material, further inquiries can be directed to the corresponding author/s.

## Ethics Statement

The studies involving human participants were reviewed and approved by the Institutional Review Board of Jeju National University Hospital. Written informed consent for participation was not required for this study in accordance with the national legislation and the institutional requirements.

## Author Contributions

J-GK and DL conceptualized and designed the study. J-GK, YS, C-HK, JC, DS, and DL reviewed relevant articles, recruited patients, and collected data. J-GK, JC, and DL analyzed the data. All authors contributed to data interpretation, write-up, editing, revision of the final manuscript, and contributed to the production of the final version of this manuscript.

## Funding

This work was supported by a research grant from Jeju National University Hospital in 2020.

## Conflict of Interest

The authors declare that the research was conducted in the absence of any commercial or financial relationships that could be construed as a potential conflict of interest.

## Publisher's Note

All claims expressed in this article are solely those of the authors and do not necessarily represent those of their affiliated organizations, or those of the publisher, the editors and the reviewers. Any product that may be evaluated in this article, or claim that may be made by its manufacturer, is not guaranteed or endorsed by the publisher.

## Abbreviations

AIS: Acute ischemic stroke
CAD: Carotid artery dissection
CI: Confidence interval
CNS: Central nervous system
CT: Computed tomography
DWI-ASPECT: Diffusion-weighted imaging–alberta stroke program early computed tomography
ECASS: European Co-operative acute stroke study
EVT: Endovascular therapy
FLAIR: Fluid-attenuated inversion recovery
HARM: Hyperintense acute reperfusion marker
ILVO: Intracranial large vessel occlusion
IQR: Interquartile range
MRI: Magnetic resonance imaging
mRS: Modified rankin scale
NIHSS: National institutes of health stroke scale.
